# Rate of abnormalities in quantitative MR neuroimaging of persons with chronic traumatic brain injury

**DOI:** 10.1186/s12883-024-03745-6

**Published:** 2024-07-05

**Authors:** Farzaneh Rahmani, Richard D. Batson, Alexandra Zimmerman, Samir Reddigari, Erin D. Bigler, Shawn C. Lanning, Eveline Ilasa, Jordan H. Grafman, Hanzhang Lu, Alexander P. Lin, Cyrus A. Raji

**Affiliations:** 1 Department of Radiology, Washington University School of Medicine, Saint Louis, MO USA; 2 Endocrine & Brain Injury Research Alliance, Neurevolution Medicine, PLLC, NUNM Helfgott Research Institute, Portland, Oregon USA; 3 BrainSpec, Inc, Boston, MA USA; 4 Department of Neurology, Department of Psychiatry, University of Utah, Salt Lake City, UT USA; 5 Swedish Radia, Seattle, WA USA; 6 Departments of Physical Medicine & Rehabilitation, Neurology, Cognitive Neurology and Alzheimer’s Center, Department of Psychiatry, Feinberg School of Medicine, Department of Psychology, Weinberg College of Arts and Sciences, Northwestern University, Chicago, IL USA; 7 Department of Radiology, Johns Hopkins University School of Medicine, Baltimore, MD USA; 8 Center for Clinical Spectroscopy, Department of Radiology, Brigham and Women’s Hospital, Harvard Medical School, Boston, MA USA; 9 Department of Neurology, Washington University School of Medicine, Saint Louis, MO USA

**Keywords:** Traumatic brain Injury, Diffusion Tensor Imaging, Pseudocontinuous arterial spin labeling, Magnetic resonance spectroscopy

## Abstract

**Background:**

Mild traumatic brain injury (mTBI) can result in lasting brain damage that is often too subtle to detect by qualitative visual inspection on conventional MR imaging. Although a number of FDA-cleared MR neuroimaging tools have demonstrated changes associated with mTBI, they are still under-utilized in clinical practice.

**Methods:**

We investigated a group of 65 individuals with predominantly mTBI (60 mTBI, 48 due to motor-vehicle collision, mean age 47 ± 13 years, 27 men and 38 women) with MR neuroimaging performed in a median of 37 months post-injury. We evaluated abnormalities in brain volumetry including analysis of left-right asymmetry by quantitative volumetric analysis, cerebral perfusion by pseudo-continuous arterial spin labeling (PCASL), white matter microstructure by diffusion tensor imaging (DTI), and neurometabolites via magnetic resonance spectroscopy (MRS).

**Results:**

All participants demonstrated atrophy in at least one lobar structure or increased lateral ventricular volume. The globus pallidi and cerebellar grey matter were most likely to demonstrate atrophy and asymmetry. Perfusion imaging revealed significant reductions of cerebral blood flow in both occipital and right frontoparietal regions. Diffusion abnormalities were relatively less common though a subset analysis of participants with higher resolution DTI demonstrated additional abnormalities. All participants showed abnormal levels on at least one brain metabolite, most commonly in choline and N-acetylaspartate.

**Conclusion:**

We demonstrate the presence of coup-contrecoup perfusion injury patterns, widespread atrophy, regional brain volume asymmetry, and metabolic aberrations as sensitive markers of chronic mTBI sequelae. Our findings expand the historic focus on quantitative imaging of mTBI with DTI by highlighting the complementary importance of volumetry, arterial spin labeling perfusion and magnetic resonance spectroscopy neurometabolite analyses in the evaluation of chronic mTBI.

**Supplementary Information:**

The online version contains supplementary material available at 10.1186/s12883-024-03745-6.

## Introduction

Over 2.5 million U.S. citizens visit emergency departments for traumatic brain injury annually, among which over 220,000 are hospitalized [1–3]. Many suffering from TBI do not seek or receive medical care, leading to underestimation of the true prevalence of TBI [1]. The majority (70–90%), of these individuals represent mild TBI (mTBI) and up to 40% progress to develop persistent cognitive, psychosocial, and behavioral symptoms often referred to as post-concussion syndrome (PCS) [4–6]. PCS has high morbidity with reduced quality-of-life, depression and anxiety and high suicide rates [7–9]. Additionally, TBI is an independent risk factor for dementia with one study of over 350,000 participants showing a greater than two-fold increased risk even with no loss of consciousness [10].

Non-contrast head computed tomography (CT) is the standard of care for identifying life-threatening injuries from acute TBI such as hemorrhage, contusion and mass-effect [11]. CT can thus differentiate between moderate to severe and mild forms of TBI [12, 13]. Addition of non-contrast magnetic resonance imaging (MRI) improves upon the detection sensitivity for contusions, brainstem injuries and axonal injuries specially in moderate to severe TBI [14]. However, only 5–49% of patients with mTBI and negative CT scan have abnormal findings on conventional MRI sequences such T1- T2 and diffusion weighted imaging [15–18]. Absence of reliable visual findings on conventional MRI add to the challenge of objectively identifying brain damage in acute mTBI [19].

An array of FDA-cleared neuroimaging processing tools can offer crucial diagnostic insights into mTBI, beyond conventional qualitative imaging reads, by quantitatively assessing regional brain volumes, cerebral perfusion, white matter (WM) structure, and neurometabolites [20–24]. Post-traumatic cortical atrophy is thought to originate from widespread WM injury and subsequent cortical Wallerian degeneration, progressing over time and space [25]. Meta-analytic evaluation of volumetric studies have shown the thalami, temporal and frontal lobes, and cortical WM as signature atrophy regions of chronic mTBI [26]. Disruption in WM is evidenced by changes in diffusion tensor parameters namely changes in fractional anisotropy (FA) [23]. Perfusion imaging abnormalities in TBI build on the understanding that acute trauma can disrupt cerebral perfusion autoregulation and the integrity of the blood-brain barrier hence leading to chronic hypoperfusion [27, 28]. Magnetic resonance spectroscopy (MRS) is a well-known technique to assess changes in neuronal metabolites with emerging application in TBI. Growing availability of FDA-cleared software to interpret these quantitative MR neuroimaging techniques enables the application of these tools in high-throughput clinical settings [29]. With close to 40 million MRI examinations performed in the United States annually, mostly neuroradiological studies [30], there is a significant opportunity to leverage these processing tools to gain key insights that directly impact clinical decision-making.

We investigated the frequency of abnormal quantitative findings of brain regional volumes, cerebral perfusion with ASL, WM microstructure with diffusion tensor imaging (DTI) and brain metabolites with MRS using predominantly FDA-cleared image processing tools in a cohort primarily composed of individuals with mTBI. We tested the hypothesis that these quantitative neuroimaging findings would be more sensitive in reflecting TBI than conventional qualitative radiology reads. Secondarily, we compared these quantitative measurements across motor vehicle collision (MVC) versus non-MVC participants, as MVC is the most common mechanism of mTBI [31, 32] and constituted the majority of our sample. Results of this research would be pivotal for establishing the spectrum of expected quantitative imaging abnormalities using increasingly available FDA-cleared tools when evaluating a patient with mTBI.

## Methods

### Participants

We obtained cross-sectional data in 65 individuals with a history of TBI who were referred to Neurevolution Medicine, a private medical clinic focusing on brain injury medical care and forensics, between February 2018 and May 2023. This was done through consecutive convenience sampling under IRB exemption#Pro00071328. Eligibility criteria were: (1) have a history of external force to head confirmed through review of medical records available at or around the time of the traumatic event and (2) brain MRI at any time post injury that included at least a 3D T1-weighted scan. Participants were excluded if they had a history of severe, pre-existing neurological disorder or a documented history of severe mental illness including major depressive disorder, schizophrenia, posttraumatic stress disorder, or bipolar disorder. Mechanism of injury including MVC and non-MVC accidents and severity of TBI based on standard classifications were utilized [20–22, 33]. MR neuroimaging was acquired more than 6 months following injury in the chronic post-injury phase in all participants (median(Q1-Q3): 37(27–45) months). The clinical reads were provided by a board-certified neuroradiologist with approximately 10 years of experience. For the purposes of this study, a positive clinical read indicative of TBI was defined as any radiological finding where trauma was attributed to the findings in the impression section of the report, either as the primary consideration or within the provided differential diagnoses.

### MR image acquisition

MR imaging of all participants was performed on a 3 Tesla GE Signa HDx scanner. T1-weighted scans were all acquired through fast spoiled gradient echo (FSPGR) using a 32-channel head coil (software version DV26, GE HealthCare, Chicago, IL). Additional T1-weighted image acquisition parameters included: slice thickness = 1 mm, TR = 6.94 ms, TE = 3.03 ms, flip angle = 8°, FOV = 256 × 256 mm^2^, and voxel size = 1 × 1 × 1 mm^3^.

Diffusion tensor imaging (DTI) scan that was acquired through echo-planar imaging and using the following parameters: 24 directions, voxel size = 1.5 × 1.5 × 3 mm^3^, slice thickness = 1.5 mm, TR = 8000 ms, TE = 83.3 ms, and with b-value = 1000 s/mm2. Among these, twenty-one individuals had an additional DTI acquisition with the following parameters: 30 directions, isotropic 2 × 2 × 2 mm^3^ voxel size, 2 mm slice thickness, and TR = 16,716 ms, TE = 78.7 ms. This additional DTI acquisition allowed for additional quantitative assessment of these participants through the Advanced Neuro Diagnostic Imaging (ANDI) tool [34].

Perfusion imaging was performed via a 3D background-suppressed pseudo-continuous arterial spin labeling (pCASL), herein and after abbreviated as ASL, with the following parameters: single-shot gradient-echo EPI, FOV = 128 × 128, slice thickness = 4 mm, labeling duration = 1450 ms, post labeling delay = 2025 ms, TR = 4844 ms, TE = 10.5 ms, number of controls/labels = 40 pairs, labeling pulse flip angle = 90°, and perfusion calibration images (M0) for basic tissue magnetization to determine absolute cerebral blood flow [35].

Finally, to obtain proton magnetic resonance spectroscopy (^1^H-MRS) imaging, a single-voxel point-resolved spin echo (PRESS) sequence with TR = 2000 ms and TE = 30 ms (5000 Hz bandwidth with 4096 points, 64–128 averages) was applied to a 2 × 2 × 2 cm voxel in the posterior white matter as the region of interest, using the grey matter on the T1-coronal images as a guide. The PRESS acquisition parameters were as follows: total number of acquisitions = 96, averages per acquisition = 8, total number of spectra = 6, shimming method = manual shimming of water and shimming threshold = less than 14 Hz [36, 37].

Importantly, the DTI and MRS had not been performed for all participants, yielding to smaller sample sizes for these two modalities respectively (*n* = 57 and *n* = 42 for DTI and MRS: respectively).

### Quantitative image post processing

#### Volumetric processing

All participants’ 3D T1-weighted images were inputted into volumetric analyses using the FDA-cleared NeuroReader online platform (BrainReader ApS, Version 2.7). The NeuroReader (NR) and asymmetry indices (AI) were extracted for 26 distinct cortical and subcortical structures [38]. The NR-index is calculated by dividing z-scores for normalized regional volumes over the square root of the size of NeuroReader normative database for participant age and sex group: hence improving upon conventional z-scores by accounting for variabilities in group-specific sample size in the normative cohort. This normative database comprised of 231 participants (48.5% women) aged between 60 and 90 years and was derived from the Alzheimer’s Disease Neuroimaging Initiative grand opportunities (ADNI-GO) study [39]. The AI is defined as the ratio of left-right volume difference divided over the total volume of any given region, where an AI of above zero indicates left > right asymmetry [22]. The NR-index for AI was calculated similarly to the NR-index for normalized regional volumes. The NR-indices were noted abnormal if below or equal to -2 in lobar and subcortical gray matter (GM) structures or if above or equal to + 2 in the lateral ventricles, based on prior published work on the application of Neuroreader in TBI [22]. Segmentation maps were meticulously reviewed by a board certified neuroradiologist experienced in quantitative neuroimaging analysis (CAR) to identify any significant errors that could lead to inaccurate measurements of the NR or AI indices.

#### Cerebral perfusion analyses

Quantitative Perfusion analyses was performed in all participants using the ASL-MRICloud to extract relative cerebral blood flow (rCBF) [40]. The ASL-MRICloud is an online tool that generates rCBF for 287 regions that are compared to their age and sex-matched individuals from a healthy normative database to generate z-score maps, as described in prior work [41]. The normative database used by the ASL-MRICloud comprised of 309 healthy volunteers ranging from 20 to 89 years old (48.7% women) who were evenly distributed across 5-year age brackets [42]. Only z-score maps, and not regional z-scores, were provided as part of the standard ASL-MRICloud output, which prevented us from performing quantitative comparisons based on regional rCBF Z-scores. ASL-MRICloud also conducts automated image quality indices of processed CBF maps on a scale of 1–4 (1 = Excellent, 4 = Poor). All maps received a score of 1 from ASL-MRICloud and were confirmed on separate visual inspection by a neuroradiologist experienced in both ASL and quantitative neuroimaging (CAR).

#### Diffusion tensor imaging

DTI sequences were available through the proprietary Swedish-Radia pipeline for 57 participants yielding FA maps from diffusion-weighted images. A subgroup of 21 participants who had additional DTI sequences compatible with the FDA-approved Advanced Neuro Diagnostic Imaging (ANDI) tool (Imeka Solutions Inc., Quebec, Canada) [34, 43–45] were additionally analyzed through this pipeline. The ANDI requires DTI data acquired to certain protocol specifications as detailed above, limiting the number of participants in this subsample.

In the Swedish-Radia pipeline, a software package (Nordic ICE Diffusion/DTI Module, Nordic Imaging Laboratory, Bergen, Norway) was used to generate DTI FA maps and z-scores compared to Swedish-Radia normative data for 8 hand drawn ROIs located in the frontal and parietal WM, periventricular WM, the genu and splenium of corpus callosum, and internal capsules [46]. In order to calculate the z-scores, each participant is compared to their respective 10-year age bracket from the Swedish-Radia normative dataset (example; the z-score for a 45-year-old participant was calculated by taking the difference between their DTI parameter and the average DTI parameter for the normative dataset participants aged 40–49 years, and then dividing this difference by the standard deviation of that parameter within the same age group). The mentioned normative dataset was composed of 80 healthy volunteers who were imaged using the same acquisition parameters and scanner (age range 1 to 79 years old, about 10 control participants for each decade). Abnormal FA z-scores were defined as either below a cut-off of at, or less then − 2 or greater than or equal to + 2 based on standard thresholds used by Swedish and as detailed in prior peer-reviewed literature [47, 48]. Experienced MR technicians, SCL and EI reviewed the ROIs and related FA and z-score calculations for quality assurance.

In ANDI, a whole-brain reconstruction WM algorithm leverages reinforcement learning to generate a tractogram using the fiber orientation density function (fODF). This process begins by preprocessing the diffusion-weighted images to correct for motion, eddy currents, and susceptibility artifacts, as well as performing N4 bias correction. Next, the DWIs are resampled into the T1 image space, and the T1 image is registered to the DWI image to generate seeding/anatomic masks. A stream-line probabilistic-based tractography method is then used to generate tracts from the previously computed axial diffusivity (AD), fractional anisotropy (FA), mean diffusivity (MD), and radial diffusivity (RD) maps for 33 principal WM bundles. Lastly, the mean DTI-derived measures and their respective confidence interval measurements were generated for each tract bundle and compared to the ANDI representative normative reference to generate tract-specific z-scores [49, 50]. The normative dataset is comprised of a balanced cohort of 1266 healthy individuals (51.3% females), ranging in age from 20 to 80 years, stratified evenly across 10-year age brackets [49]. Similar to Swedish-Radia ROI approach, participants were matched to their corresponding 10-year age bracket to calculate the z-scores. DTI values below 5th or above 95th confidence intervals for FA, AD, MD and RD were considered abnormal, as noted in the ANDI manual.

#### Proton magnetic resonance spectroscopy

MRS studies had been obtained on 42 participants and processed using the web-based FDA cleared BrainSpec software to measure concentrations of different neurometabolites (https://www.brainspec.co) [51]. Neurometabolites included N-acetylaspartate (NAA), a marker of neuronal density and viability, choline (Cho), a marker of membrane turnover and cellular proliferation, glutamate (Glx), a marker of excitotoxicity, lactate (Lac), a marker of anaerobic cellular metabolism, and myoinositol (mI), a marker of glial cell proliferation such as in neuroinflammation [52, 53]. Creatine (Cr) which is found in metabolically active tissues was used as an internal reference standard for other metabolites as the output measure (e.g., NAA/creatine) [52]. The region of interest was set to the left posterior white matter as it is a region that has been shown to be sensitive to brain injury [21]. A minimum signal-to-noise ratio (SNR) of 10 was considered as satisfactory quality of the MRS and the spectra of all patients passed this threshold with a mean SNR of 21 (range 11–31). A maximum linewidth of 0.1 ppm was considered as satisfactory quality, for which the spectra of all patients passed (mean linewidth: 0.03 ppm, range: 0.024–0.067 ppm). In addition, the reliability of each metabolite measurement was assessed by the Cramer-Rao lower bound (CRLB) as a function of the metabolite concentration but was not used for data filtering [54]. The mean (range) CRLB of NAA, Cr, Cho, and Glx were 2.8% (2–4), 2,9% (2–4), 4.8% (3–7), and 7.9% (4–14) respectively. Output measures below or above the reference range were considered abnormal as specified by the BrainSpec software.

### Statistical analyses

Analyses were all done using the R software version 4.0.5 (https://www.r-project.org/). We used the *shapiro.test* function from the statistical package to investigate the normality assumption for the residuals of variables. The *chisq.test* with or without the Fisher’s exact function were used to compare the frequency of abnormal NR-indices, neurometabolite abnormalities, sex, and severity of TBI, between the MVC and non-MVC groups. The average NR-indices, AD, FA, MD, RD, age, or time to MR imaging, were compared using the *Mann-Whitney U test*. Given the low frequency and sample size of DTI abnormalities using either the Swedish-Radia or ANDI datasets, between group comparisons were not performed for DTI abnormalities. Multiple comparisons correction was done using the Benjamini-Hochberg false-discovery rate (FDR) [55].

## Results

**Fig. 1:**
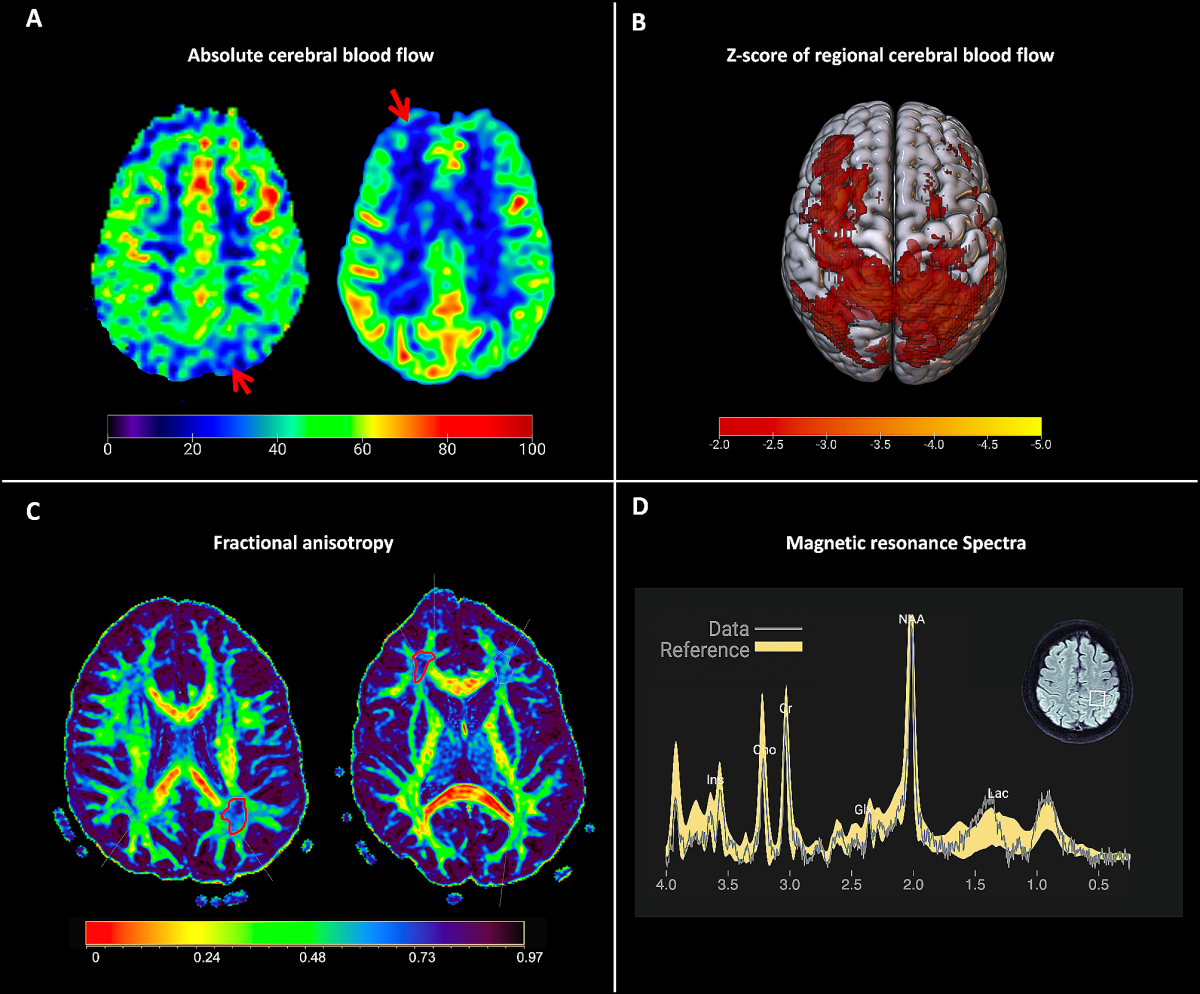
Examples of participants with abnormal cortical perfusion, white matter diffusion and neurometabolites. **A**: reduced absolute cerebral blood flow in a coup-countercoup pattern (red arrow heads) in a 40-year-old man with blunt object injury to the right face. Images acquired 30 months post-injury, **B**: Abnormal z-scores (<-2) in regional blood flow maps of the same participant demonstrating a similar pattern, **C**: fractional anisotropy maps in a 23-year-old man with moderate to severe TBI following MVC. Regions of interest (red delineations) point to areas with low z-score in the left parietal (-2.2) and right frontal (-2.6) WM, calculated separately based on age and sex-matched reference ranges. Images acquired 23 months post-injury, **D**: MR spectra of a 67-year-old female with mild TBI following rear-ended MVC demonstrating the observed spectrum from the patient in grey and age and sex-matched reference spectra in yellow. Reference voxel shown as white box in the top right axial image. This participants NAA/Cr, Cho/Cr and mI/Cr were above, and Glx/Cr was below the reference range. Images were acquired 47 months post-injury. Abbreviations: TBI: traumatic brain injury; MVC: motor-vehicle collision; WM: white matter

Table 1 summarizes the demographic and clinical information of the entire study population. Participants with MVC did not differ in their age, sex, severity of TBI or the time interval between injury to MR imaging when compared with those with non-MVC mechanism of injury. In 75.4% of participants there were no finding in the clinical read that was positively attributed to TBI, consistent with the fact that the majority of participants (92.3%) were clinically diagnosed with mTBI. Figure 1 demonstrates examples of abnormal perfusion, diffusion and neurometabolite findings.

**Table 1:** Clinical features of study participants with MVC versus non-MVC mechanism of injury

	Total (*n* = 65)	MVC (*n* = 48)	Non-MVC (*n* = 17)	*P*-value*
Age, years (mean±sd)	47±14	45±13	50±14	0.1
Sex, men/women, n(%)	27(41.5%)/38(58.5%)	18(37.5)/30(62.5)	9(53)/8(47)	0.2
Severity of TBI, n(%)	mild: 60(92.3) moderate: 4(6.2) severe: 1(1.5)	mild: 43(89.6) moderate: 4(8.4) severe: 1(2)	mild: 17(100)	0.057
Mechanism of injury, n(%)	**-**	Rear-end (15(31.2)) Frontal crash (8(16.7)) T-bone (4(8.4)) Rolled-over (3(6.2)) NS (18(37.5))	Blunt object injury (9(53)) Fall (6(35.2)) Blast (1(5.9)) NS (1(5.9))	**-**
Time to advanced MRI, months (median(Q1-Q3))	37(27–45)	38(23–48)	30(27–39)	0.2
Positive visual read for TBI, n(%)	16(24.6%)	12(25%)	4(23.5%)	0.2

Abbreviations: TBI: traumatic brain injury; MVC: motor vehicle collision; non-MVC: mechanism other than motor vehicle collision; NS: Not specified; Time to MRI: time-lapse between injury and advanced MRI acquisition that was used for current analyses, Q1 and Q3: quartiles 1 and 3

*P-value of the students T-test or Mann-Whitney U test for variables with and without normal distribution

### Volumetric analyses

All participants had abnormal NR-index in at least one lobar (frontal, parietal, occipital or temporal) white or gray matter volume or lateral ventricular volumes. There were no statistically significant differences in the regional NR-indices between MVC and non-MVC patients (*p*-value of all tests > 0.05 after FDR correction). Atrophy in the cerebellar GM, temporal lobe, pallidi, ventral diencephalon, and lateral ventricular enlargement were the most common NR-index abnormalities (Table 2). Those with MVC mechanism demonstrated lower NR-indices for left-right asymmetry in the frontal lobe GM (*p*-value:0.031) and temporal lobe WM (*p*-value:0.017), indicating more pronounced right > left asymmetry in the frontal lobe GM and in the temporal lobe WM in MVC compared to the non-MVC group (Table 3).

**Table 2:** Brain regions with abnormal NR-Index separated by mechanism of TBI

	MVC		Non-MVC		Total		*P*-value**	
	Right*	Left*	Right	Left	Right	Left	Right	Left
Hippocampus	20(41.6)	17(35.4)	8(47.1)	9(52.9)	28(43)	26(40)	0.9	0.6
Amygdala	29(60.4)	15(31.2)	7(41.2)	5(29.4)	36(55.3)	20(30.7)	0.6	0.6
Putamen	4(8.3)	4(8.3)	3(17.6)	3(17.6)	7(10.7)	7(10.7)	0.2	0.2
Thalamus	11(22.9)	8(16.6)	5(29.4)	4(23.5)	16(24.6)	12(18.4)	0.3	0.7
Ventral Diencephalon	18(37.5)	22(45.8)	10(58.8)	12(70.6)	28(43)	34(52.3)	0.9	0.7
Pallidum	34(70.8)	33(68.7)	14(82.4)	14(82.4)	48(73.8)	47(72.3)	0.9	0.9
Caudate	0(0)	0(0)	1(5.9)	0(0)	1(1.5)	0(0)	0.9	0.9
Cerebellum	14(29.1)	17(35.4)	10(58.8)	8(47.1)	24(36.9)	25(38.4)	0.6	0.9
Cerebellar GM	42(87.5)	43(89.5)	16(94.1)	16(94.1)	58(89.2)	59(90.7)	0.9	0.5
Cerebellar WM	0(0)	1(2)	0(0)	0(0)	0(0)	1(1.5)	0.9	0.9
Cerebrum	20(41.6)	20(41.6)	9(52.9)	7(41.2)	29(44.6)	27(41.5)	0.8	0.9
Cerebral GM	12(25)	12(25)	7(41.2)	4(23.5)	19(29.2)	16(24.6)	0.9	0.9
Cerebral WM	17(35.4)	20(41.6)	5(29.4)	6(35.3)	22(33.8)	26(40)	0.9	0.9
Frontal Lobe	14(29.1)	17(35.4)	8(47.1)	7(41.2)	22(33.8)	24(36.9)	0.9	0.6
Frontal Lobe GM	9(18.7)	8(16.6)	6(35.3)	3(17.6)	15(23)	11(16.9)	0.6	0.1
Frontal Lobe WM	16(33.3)	24(50)	4(23.5)	6(35.3)	20(30.7)	30(46.1)	0.9	0.8
Occipital Lobe	14(29.1)	15(31.2)	8(47.1)	5(29.4)	22(33.8)	20(30.7)	0.6	0.055
Occipital Lobe GM	25(52)	19(39.5)	9(52.9)	9(52.9)	34(52.3)	28(43)	0.2	0.2
Occipital Lobe WM	8(16.6)	8(16.6)	3(17.6)	3(17.6)	11(16.9)	11(16.9)	0.9	0.2
Parietal Lobe	9(18.7)	13(27)	6(35.3)	7(41.2)	15(23)	20(30.7)	0.9	0.4
Parietal Lobe GM	7(14.5)	12(25)	7(41.2)	8(47.1)	14(21.5)	20(30.7)	0.3	0.1
Parietal Lobe WM	10(20.8)	9(18.7)	6(35.3)	5(29.4)	16(24.6)	14(21.5)	0.8	0.7
Temporal Lobe	32(66.6)	27(56.2)	12(70.6)	8(47.1)	44(67.6)	35(53.8)	0.9	0.8
Temporal Lobe GM	20(41.6)	13(27)	11(64.7)	6(35.3)	31(47.6)	19(29.2)	0.8	0.6
Temporal Lobe WM	27(56.2)	29(60.4)	7(41.2)	6(35.3)	34(52.3)	35(53.8)	0.7	0.3
Lateral Ventricle	33(68.7)	32(66.6)	10(58.8)	8(47.1)	43(66.1)	40(61.5)	0.4	0.2

* All frequencies are demonstrated as number (*n*) and percentage (%) within group

** Mann-Whitney U *p*-value

Abbreviations: NR-index: Neuroreader Index; MVC: motor-vehicle collision; GM: gray matter; WM: white matter; TBI: traumatic brain injury

**Table 3:** Frequency of participants with abnormal NR-Index for AI separated by mechanism of TBI

	MVC, *n*(%)*	Non-MVC, *n*(%)*	Total, *n*(%)*	*P*-value**
Hippocampus	15(31.2)	4(23.5)	19(29.2)	0.06
Amygdala	24(50)	10(58.8)	34(52.3)	0.9
Putamen	23(47.9)	8(47.1)	31(47.6)	0.7
Thalamus	19(39.5)	11(64.7)	30(46.1)	0.5
Ventral Diencephalon	26(54.1)	7(41.2)	33(50.7)	0.7
Pallidum	32(66.6)	12(70.6)	44(67.6)	0.4
Caudate	22(45.8)	12(70.6)	34(52.3)	0.4
Cerebellar GM	6(12.5)	3(17.6)	9(13.8)	0.5
Cerebellar WM	7(14.5)	3(17.6)	10(15.3)	0.9
Cerebellum	25(52)	11(64.7)	36(55.3)	0.6
Cerebral GM	11(22.9)	7(41.2)	18(27.6)	0.5
Cerebral WM	10(20.8)	5(29.4)	17(26.1)	0.5
Cerebrum	20(41.6)	6(35.3)	26(40)	0.5
Frontal Lobe	24(50)	7(41.2)	31(47.6)	0.3
Frontal Lobe GM	14(29.1)	10(58.8)	24(36.9)	***0.034***
Frontal Lobe WM	24(50)	9(52.9)	33(50.7)	0.4
Lateral Ventricle	24(50)	6(35.3)	30(46.1)	0.7
Occipital Lobe	25(52)	7(41.2)	32(49.2)	0.9
Occipital Lobe GM	20(41.6)	7(41.2)	27(41.5)	0.7
Occipital Lobe WM	18(37.5)	9(52.9)	27(41.5)	0.3
Parietal Lobe	18(37.5)	7(41.2)	25(38.4)	0.4
Parietal Lobe GM	8(16.6)	6(35.3)	14(21.5)	0.1
Parietal Lobe WM	9(18.7)	3(17.6)	10(15.3)	0.7
Temporal Lobe	19(39.5)	11(64.7)	30(46.1)	0.8
Temporal Lobe GM	16(33.3)	5(29.4)	21(32.3)	0.9
Temporal Lobe WM	8(16.6)	8(47.1)	16(24.6)	***0.017***

* Frequencies are demonstrated as number (*n*) and percentage (%) within group

**T-test or Mann-Whitney U test *P*-value where appropriate

Abbreviations: NR-index: Neuroreader Index; AI: asymmetry index; MVC: motor-vehicle collision; GM: gray matter; WM: white matter; TBI: traumatic brain injury

**Table 4:** Frequency of abnormal brain metabolites in the studies population separated by mechanism of TBI

	**MVC (** *n* ** = 33)**			**Non-MVC (** *n* ** = 9)**			**Total (** *n* ** = 42)**			*P*-value**
	**Normal, ** *n* (%)*	**Decreased, **/*n*(%)*	**Increased, ** *n* (%)*	**Normal, ** *n* (%)*	**Decreased, ** *n* (%)*	**Increased, ** *n* (%)*	**Normal, ** *n* (%)*	**Decreased, ** *n* (%)*	**Increased, ** *n* (%)*	
NAA/Cr	20(60.6)	3(9.1)	10(30.3)	7(77.8)	0(0.0)	2(22.2)	27(64.3)	3(7.1)	12(28.6)	0.6
Cho/Cr	14(42.2)	18(54.5)	1(3.1)	4(44.4)	5(55.6)	0(0)	18(42.9)	23(54.8)	1(2.4)	0.8
Glx/Cr	14(42.4)	2(6.1)	17(51.5)	2(22.2)	0(0)	7(77.8)	16(38.1)	2(4.8)	24(57.1)	0.2
Lac/Cr	32(97)	0(0)	1(3)	9(100)	0(0)	0(0)	41(97.6)	0(0)	1(2.4)	0.8
mI/Cr	21(63.6)	0(0)	12(36.4)	5(55.6)	0(0)	4(44.4)	26(61.9)	0(0)	16(38.1)	0.6

* Frequencies are demonstrated as number (*n*) and percentage (%) within group

**T-test or Mann-Whitney U test *P*-value where appropriate

Abbreviations: MVC: motor-vehicle collision; NAA: N-Acetylaspartate; Cho: Choline; Glx: Glutamate/Glutamine; Lac: Lactate; TBI: traumatic brain injury; mI: myoinositiol

### Perfusion imaging

**Fig. 2:**
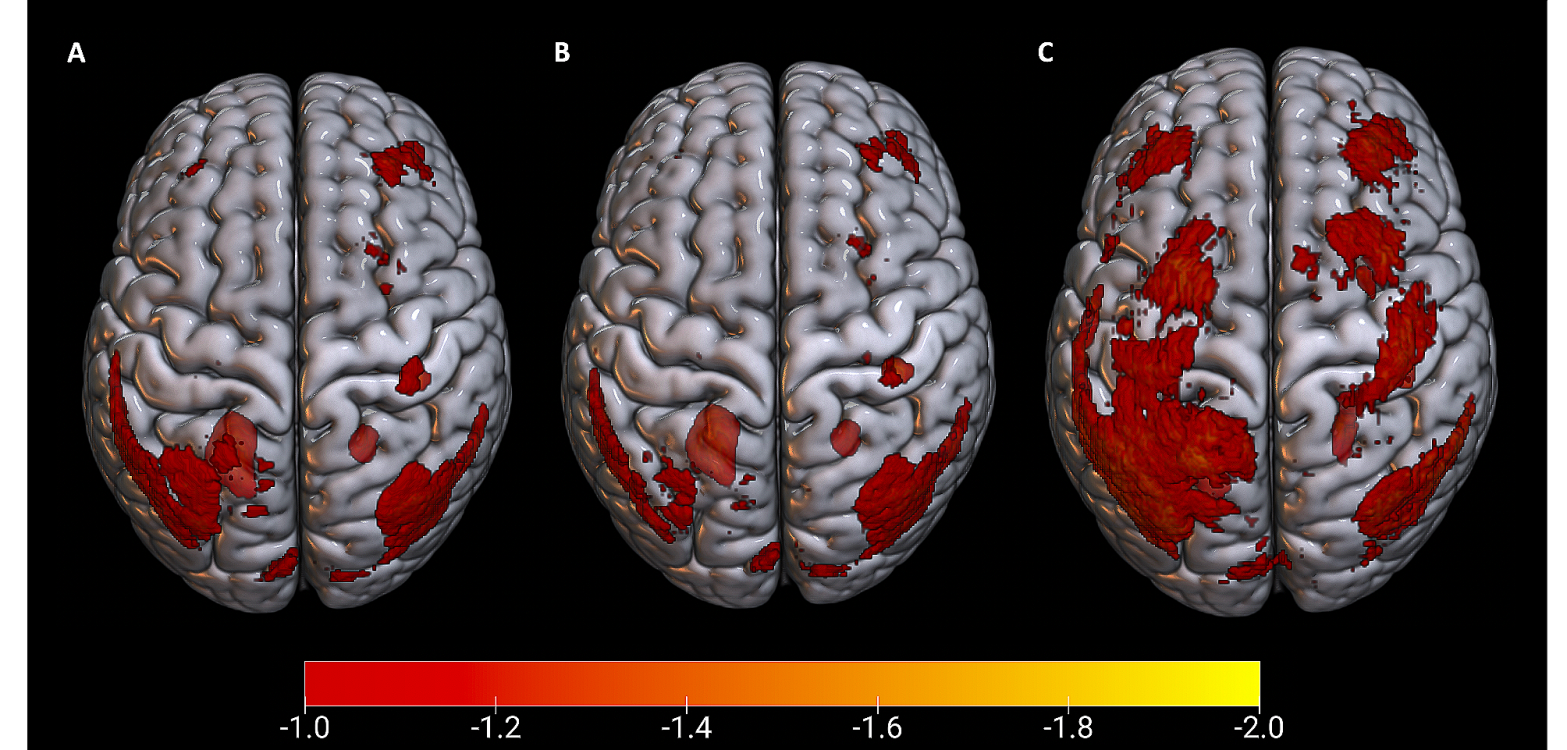
Average rCBF z-score maps in individuals with a history TBI. **A**: Average maps across entire sample reflecting a coup-contrecoup mechanism with asymmetric frontal hypoperfusion and contralateral posterior cortical low CBF; **B**: across individuals who sustained MVC suggesting a coup-contrecoup mechanism given the asymmetric frontal lobe decreased CBF with contralateral additional posterior cortical hypoperfusion; and **C**: across individuals with non-MVC mechanism of injury. All maps were thresholded to only reflect areas with z-score < -2 prior to averaging. For visualization purposes the scale is set to demonstrate z-scores between − 1 and − 2. Abbreviations: rCBF: relative regional blood flow; TBI: traumatic brain injury; MVC: motor-vehicle collision

Figure 2 demonstrates average rCBF z-score maps across the entire population, and in the MVC and non-MVC groups separately. Individual z-score maps were thresholded to only reflect voxels with z-score below or equal to -2 in adherence with prior peer-reviewed studies on the application of z-score maps to perfusion neuroimaging evaluations [56, 57]. In the ASL scans of this cohort, 100% of those scans had abnormal z-score maps. Perfusion abnormalities in the right frontal and left occipital cortices as seen in Fig. 2, most pronounced in the entire sample (part A) and the MVC group (Part B), are compatible with a coup-contrecoup injury mechanism. Part C reflects non-MVC mechanism as the frontal and posterior cortical hypoperfusion are comparatively bilateral and symmetric compared to the MVC pattern in Part B. When compared between the MVC versus non-MVC group there was no statistically significant difference in rCBF in any of the 287 investigated cortical and subcortical regions after accounting for multiple comparisons (*p*-value of all regions < 0.05).

### Diffusion tensor imaging

Using ROIs from Swedish-Radia, only a small percentage of patients exhibited abnormally low FA z-scores. These included 6% (*n* = 4) and 7.7% (*n* = 5) of participants, all demonstrating FA z-scores at or below − 2, in the right and left internal capsules, respectively. Only one participant had low FA z-score at or below − 2 in the genu and splenium of the corpus callosum or in the posterior lobar WM each. There were no statistically significant differences in the FA z-scores between individuals who sustained MVC compared with the non-MVC group (*p*-value of all tests > 0.05 after FDR correction). In terms of elevated FA, 40.7% of participants (22/54) had FA z-scores at or above + 2 in at least one ROI. Of these the left (11/22) and right parietal WM (10/22), followed by the left (3/22) and right (6/22) frontal WM and the left (5/22) and right (2/22) internal capsules were most likely to have FA z-scores at or above + 2.

Standardized AD, FA, MD, and RD values obtained from ANDI were considered out of range if < = 5^th^ and > = 95^th^ percentile, across 33 investigated WM tracts. Most WM tracts with abnormality demonstrated elevated (above 95 percentile) DTI metrics, more commonly FA and AD (Supplementary Table 1). Among abnormal tracts both arcuate fasciculi, cingulate gyri, frontal aslant occipital tracts, fornices, and mid genu of the corpus callosum were most likely to demonstrate abnormally elevated AD and FA.

### Neurometabolite analyses

In total, 38 out of 42 participants with MRS (90.4%) data had abnormal levels of at least one of the investigated metabolites; NAA/Cr, Cho/Cr, Glx/Cr, lac/Cr, mI/Cr, of which 28/38 had more than one metabolite above or below the normal reference range (Table 4). Table 4 summarizes the frequency of abnormal metabolite findings from MRS in the study participants.

## Discussion

The use of quantitative approaches to objectively identify abnormalities on neuroimaging dates back to Minoshima et al.’s seminal paper from 1995 demonstrating the use of Z-score maps with 3D-SSP to demonstrate abnormally low z-scores in single patient comparisons to normative data [58, 59]. This method of voxel based statistical calculations on individual neuroimaging data compared to normative datasets has subsequently informed development of the FDA cleared programs used in this work including Neuroreader, ANDI, and BrainSpec. Official guidelines from the American College of Radiology on the application of advanced neuroimaging in TBI are almost a decade old and do not reflect the comparatively recent innovations of these FDA cleared programs [60]. While the ASL program used in this work, ASL-MRICloud, is not FDA cleared it also uses a similar approach as 3D-SSP and encourages the development of ASL analysis tools that hold promise for broader clinical applications [61–63]. Although the methods employed in this paper are based on validated approaches that have been established for several decades, their adoption in clinical practice remains limited. For example, a commonly used FDA-cleared volumetric tool in 2017 was estimated to have generated 175,000 reports across 200 institutions [64]. Even if all of these reports were generated within a single year, they would account for only 0.4% of the total 40 million MRI scans performed annually [30], the majority of which are neuroradiology based. Thus, additional work remains to be done to integrate quantitative neuroimaging into clinical practice, for techniques other than DTI, which is the most commonly applied method for quantifying TBI abnormalities [65]. Our work suggests that such application is feasible given the increased availability of FDA-cleared quantitative neuroimaging tools and their enhanced ability to identify subtle abnormalities in TBI.

We found a notably high prevalence of abnormal findings using quantitative neuroimaging techniques in a mTBI predominant group. All of our participants showed atrophy and diminished perfusion in at least one lobar structure and in approximately two thirds of the participants there was significant left-right asymmetry in at least one lobe. Among less investigated techniques, abnormal levels of at least one neurometabolites was seen in more than 90% of participants, whereas alterations in DTI metrics were relatively uncommon, ranging between < 1–17% when evaluated as either WM ROI or discrete WM tracts. When contrasted against positive visual reads, which were seen in only 24.6% of the participants, these results underscore the potential utility of quantitative imaging techniques in the diagnosis of chronic mTBI sequelae. The relative lack of visual findings aligns with previous data indicating that visual reads of conventional CT head scans are positive only in 15% of TBI cases and 34% for common clinical MRI scan sequences, such as 2D T1, T2, FLAIR, and susceptibility-sensitive or gradient echo sequences [66].

Brain atrophy in TBI is noted as early as 2–3 weeks post-injury [67]. While these atrophy patterns are well-described in moderate to severe forms of TBI, the comparative subtlety of these changes in mTBI mandates the use of quantitative volumetric processing combined with appropriate timing of the imaging for optimized sensitivity [68–70]. Also, while the predominant literature focuses on between group statistical testing, qualitative inference based upon quantitative volumetric outputs, such as NR and asymmetry indices could yield to more clinically-relevant conclusions. Using the NeuroReader platform, we demonstrate the ubiquity of such findings in a cohort predominated by chronic mTBI. These findings build upon prior data showing volume loss on NeuroReader in chronic TBI [22]. While NeuroReader has also demonstrated atrophy from other causes, such as Alzheimer’s disease [38], it is unlikely to be a confounding source of atrophy in our study due to the average midlife age of our cohort and the absence of characteristic bilaterally symmetric temporal and parietal lobe atrophy. Volumetric asymmetry is a feature of TBI due most likely to variations in TBI related forces and a compensatory neuroinflammatory response [71, 72]. Asymmetry of subcortical GM structures such as globus pallidi, amygdalae and hippocampi and the temporal and frontal cortex are reported in the literature [71, 73]. Similarly, we observed asymmetry in the volume of globus pallidi in approximately two-thirds, and in the volume of amygdalae, putamina, thalami, and caudate nuclei in approximately half of our participants.

Cerebral hypoperfusion after TBI is attributed to disruption of the neurovascular unit, impairment of cerebral autoregulation and blood brain barrier, as well as neuroinflammation that may also contribute to these findings [20, 33, 74]. Preclinical research shows a reduction in CBF in the non-injured hemisphere in the acute post-mTBI phase [28, 75, 76]. One study in patients with MVC-related mTBI demonstrated reduced CBF in both frontal and left occipital lobes [77], similar to our findings which might indicate a coup-contrecoup mechanism for perfusion abnormalities. These findings are distinct from the FDG-PET concordant precuneus and posterior cingulate hypoperfusion reported for Alzheimer disease [78]. Further research is required to elucidate the patterns of perfusion abnormalities in chronic mTBI.

Changes in DTI metrics are widely investigated in the acute post-mTBI phase and associated with long-term deficits in different cognitive domains [79, 80]. Reduced FA, increased AD, and MD of the corpus callosum, corona radiata, internal capsule, and the corticospinal tracts, cingulum, frontooccipital and longitudinal fasciculi are commonly reported in mTBI and have been shown in both ROI- and tractography-based methods [81–85]. Using an ROI-based method and cut-off values based on standardized DTI metrics, we found a relatively low frequency of abnormal FA which might reflect lower sensitivity of this approach to detect WM damage post mTBI. With tract-based statistics and within the subsample of participants processed through the ANDI pipeline we demonstrated elevated FA, and to a lesser extent elevated AD and MD, in several of the previously reported WM tracts and in up to two-thirds of this limited sample. Elevated FA, that was noted in both the Swedish-Radia and ANDI DTI FA results, has also been reported in TBI and may represent microstructural correlates of scarring and gliosis [86–89].

MRS can detect molecular changes directly resulting from the underlying tissue injury in TBI. Such abnormalities include reduced NAA indicative of neuroaxonal injury, increased Cho in the setting of membrane degradation, excitotoxicity indicated by a rise in Glx peak, increased mI with glial cell activation, or emergence of lactate peak indicative of ischemia [21, 90, 91]. NAA/Cr was decreased in more than half of the participants as reported previously [21, 90, 92]. In MRS, the frontal lobe is often chosen as the ROI for studies measuring N-acetylaspartate (NAA) concentration which exhibits a more pronounced decrease in NAA concentration compared to the parietooccipital regions, including the posterior white matter which was the ROI in our study [21]. Reductions in NAA are greatest in the acute post-injury setting and tend to normalize later, whereas our cohort was chronic TBI [21]. Related to our choline data, meta-analytic results have shown that choline increase is noted in moderate-to-severe TBI and in ROI with both GM and WM [21, 85, 93].

Our study is limited by retrospective cross-sectional study design. As a result, data on longitudinal progression of imaging findings related to TBI severity are lacking in our sample. Importantly, the age range of the normative dataset used by the NeuroReader platform did not match that of our population, potentially leading to potential underestimation of volume loss in our cohort. Future work can address this limitation using longitudinal scans comparing atrophy rates in TBI to those expected in the general population [94]. However, processing with the ASL-MRICloud, Swedish-Radia and ANDI was done through matching patients to their respective age brackets in the normative datasets potentially allowing for a higher level of accuracy. While scanner and protocol acquisition differences between individual participants and normative data may pose obstacles to interpretation, these challenges are mitigated by use of complementary quantitative imaging sequences. Thus, if an individual with TBI shows abnormal quantitative neuroimaging results across all the metrics considered – i.e. (i) structure (brain volumes), (ii) perfusion (ASL), (iii) brain connections (DTI), and (iv) brain metabolites (MRS)- such confluence makes it highly unlikely that all of these results are simultaneously artifactual. Future work can leverage artificial intelligence tools that, while not currently FDA cleared, can identify new biomarkers relevant to TBI, such as accelerated brain age and elevated Alzheimer disease risk [95–98].

## Conclusion

We demonstrated a pattern of coup-contrecoup injury in the cortical CBF in a majority mTBI participants with mostly MVC related injury. Our findings highlight the importance of utilizing complimentary quantitative MR imaging techniques such as structural volumetric analysis, ASL and MRS in the imaging of chronic TBI. This work also expands the focus and potential clinical applications of quantitative neuroimaging in TBI in addition to historically emphasized approaches with DTI.

### Electronic supplementary material

Below is the link to the electronic supplementary material.

Supplementary Material 1

## Data Availability

No datasets were generated or analysed during the current study.

## Abbreviations

TBI: Traumatic Brain Injury
DTI: Diffusion tensor imaging
pCASL: pseudo-continuous arterial spin labeling
MRS: Magnetic resonance spectroscopy
WM: White matter
mTBI: mild traumatic brain injury
MVC: Motor vehicle collision
non-MVC: non-motor vehicle collision
NAA: N-acetylaspartate
Cho: choline
Glx: Glutamate
Lac: Lactate
mI: myoinositol
Cr: Creatine
ANDI: Advanced Neuro Diagnostic Imaging
FA: Fractional anisotropy
RD: Radial diffusivity
AD: Axial diffusivity
MD: mean diffusivity
ROI: Region of interest
rCBF: Cerebral blood flow
