# Supplementary material for: Rate of abnormalities in quantitative MR neuroimaging of persons with chronic traumatic brain injury
Source: BMC Neurol. 2024 Jul 5;24:235. doi: 10.1186/s12883-024-03745-6 (PMC11225195; doi:10.1186/s12883-024-03745-6)
Supplement: Supplementary file 1 — Supplementary Material 1 [file 12883_2024_3745_MOESM1_ESM.docx]

| **Supplementary Table 1.** Frequency of abnormal diffusion metrics using the ANDI pipeline in a subgroup of 21 participants | | | | | | | | | | | | | | | | |
| --- | --- | --- | --- | --- | --- | --- | --- | --- | --- | --- | --- | --- | --- | --- | --- | --- |
|  | **AD** | | | | **FA** | | | | **MD** | | | | **RD** | | | |
|  | Right | | Left | | Right | | Left | | Right | | Left | | Right | | Left | |
| Tracts | **<5th*** | **>95th*** | **<5th** | **>95th** | **<5th** | **>95th** | **<5th** | **>95th** | **<5th** | **>95th** | **<5th** | **>95th** | **<5th** | **>95th** | **<5th** | **>95th** |
| **AF** | 0(0) | 12(57.1) | 0(0) | 6(28.5) | 0(0) | 2(9.5) | 0(0) | 3(14.2) | 0(0) | 5(23.8) | 0(0) | 4(19) | 0(0) | 0(0) | 0(0) | 1(4.7) |
| **CG** | 0(0) | 10(47.6) | 0(0) | 12(57.1) | 0(0) | 10(47.6) | 0(0) | 10(47.6) | 0(0) | 3(14.2) | 0(0) | 2(9.5) | 0(0) | 1(4.7) | 1(4.7) | 0(0) |
| **CR** | 0(0) | 2(9.5) | 0(0) | 1(4.7) | 0(0) | 4(19) | 0(0) | 4(19) | 0(0) | 1(4.7) | 0(0) | 0(0) | 0(0) | 0(0) | 1(4.7) | 0(0) |
| **FAT** | 0(0) | 14(66.6) | 0(0) | 8(38) | 0(0) | 0(0) | 0(0) | 1(4.7) | 0(0) | 10(47.6) | 0(0) | 3(14.2) | 0(0) | 5(23.8) | 0(0) | 2(9.5) |
| **FX** | 0(0) | 6(28.5) | 0(0) | 7(33.3) | 0(0) | 14(66.6) | 0(0) | 13(61.9) | 0(0) | 4(19) | 0(0) | 5(23.8) | 0(0) | 4(19) | 0(0) | 4(19) |
| **IFOF** | 0(0) | 14(66.6) | 0(0) | 4(19) | 0(0) | 8(38) | 0(0) | 5(23.8) | 0(0) | 1(4.7) | 0(0) | 0(0) | 0(0) | 1(4.7) | 1(4.7) | 0(0) |
| **ILF** | 0(0) | 8(38) | 0(0) | 3(14.2) | 0(0) | 4(19) | 0(0) | 6(28.5) | 0(0) | 1(4.7) | 0(0) | 1(4.7) | 0(0) | 0(0) | 0(0) | 1(4.7) |
| **OR** | 0(0) | 3(14.2) | 0(0) | 1(4.7) | 0(0) | 11(52.3) | 0(0) | 8(38) | 0(0) | 0(0) | 0(0) | 1(4.7) | 1(4.7) | 0(0) | 1(4.7) | 1(4.7) |
| **CST** | 0(0) | 2(9.5) | 0(0) | 1(4.7) | 0(0) | 4(19) | 0(0) | 7(33.3) | 0(0) | 0(0) | 0(0) | 0(0) | 1(4.7) | 0(0) | 0(0) | 0(0) |
| **SLF1** | 0(0) | 4(19) | 0(0) | 1(4.7) | 0(0) | 0(0) | 0(0) | 0(0) | 0(0) | 3(14.2) | 0(0) | 0(0) | 0(0) | 1(4.7) | 0(0) | 0(0) |
| **SLF2** | 0(0) | 7(33.3) | 0(0) | 2(9.5) | 0(0) | 1(4.7) | 0(0) | 2(9.5) | 0(0) | 3(14.2) | 0(0) | 1(4.7) | 0(0) | 0(0) | 0(0) | 0(0) |
| **SLF3** | 0(0) | 8(38) | 0(0) | 4(19) | 0(0) | 0(0) | 0(0) | 0(0) | 0(0) | 4(19) | 0(0) | 4(19) | 0(0) | 0(0) | 0(0) | 0(0) |
| **UF** | 0(0) | 17(80.9) | 0(0) | 10(47.6) | 0(0) | 5(23.8) | 0(0) | 9(42.8) | 0(0) | 11(52.3) | 0(0) | 4(19) | 0(0) | 4(19) | 1(4.7) | 1(4.7) |
|  | **<5th** | | **>95th** | | **<5th** | | **>95th** | | **<5th** | | **>95th** | | **<5th** | | **>95th** | |
| **CC1** | 0(0) | | 20(95.2) | | 0(0) | | 17(80.9) | | 0(0) | | 6(28.5) | | 0(0) | | 0(0) | |
| **CC2** | 0(0) | | 16(76.1) | | 0(0) | | 3(14.2) | | 0(0) | | 12(57.1) | | 0(0) | | 7(33.3) | |
| **CC3** | 0(0) | | 11(52.3) | | 0(0) | | 1(4.7) | | 0(0) | | 7(33.3) | | 0(0) | | 6(28.5) | |
| **CC4** | 0(0) | | 7(33.3) | | 0(0) | | 0(0) | | 0(0) | | 8(38) | | 0(0) | | 6(28.5) | |
| **CC5** | 0(0) | | 3(14.2) | | 0(0) | | 0(0) | | 0(0) | | 5(23.8) | | 0(0) | | 5(23.8) | |
| **CC6** | 0(0) | | 1(4.7) | | 0(0) | | 3(14.2) | | 0(0) | | 1(4.7) | | 0(0) | | 0(0) | |
| **CC7** | 0(0) | | 1(4.7) | | 0(0) | | 6(28.5) | | 0(0) | | 0(0) | | 1(4.7) | | 0(0) | |
| * All numbers are presented as n(%) of participants having abnormally low (below 5^th^ percentile for age and sex) or abnormally high (above 95^th^ percentile for age and sex) diffusion metrics in the listed white matter Tracts  *Abbreviations:* AF: arcuate fasciculus; CG: cingulate gyrus; CR: coronal radiata; FAT: frontal aslant occipital; FX: fornix; IFOF: inferior frontooccipital fasciculus; ILF: inferior longitudinal fasciculus; OR: optic radiation; CST: corticospinal tract; SLF1-3: superior longitudinal fasciculus subtracts 1-3; UF: uncinate fasciculus; CC1-7: corpus callosum anterior, middle, and posterior genu, anterior and posterior body, isthmus, and splenium; AD: axial diffusivity; FA: fractional anisotropy; MD: mean diffusivity; RD: radial diffusivity | | | | | | | | | | | | | | | | |
